# Therapeutic Potential of Anti‐Diabetes Drugs and Anti‐Dyslipidemia Drugs to Mitigate Head and Neck Cancer Risk in Metabolic Syndrome

**DOI:** 10.1111/cns.70446

**Published:** 2025-05-19

**Authors:** Sujung Yeom, Dong Hoon Lee, Juhyun Song

**Affiliations:** ^1^ Department of Otolaryngology‐Head and Neck Surgery Chonnam National University Medical School & Hwasun Hospital Hwasun Republic of Korea; ^2^ Department of Anatomy Chonnam National University Medical School Hwasun Republic of Korea

**Keywords:** anti‐diabetic drugs, anti‐dyslipidemic drugs, head and neck cancer (HNC), metabolic syndrome (Mets)

## Abstract

**Background:**

Head and neck cancer (HNC) encompasses a heterogeneous group of malignancies originating in the oral cavity, pharynx, nasopharynx, larynx, paranasal sinuses, and salivary glands. Accumulating evidence indicates that metabolic syndrome (MetS) characterized by a constellation of conditions including central adiposity, hyperglycemia, dyslipidemia, hypertension, and insulin resistance, may significantly influence cancer pathogenesis and progression.

**Results:**

MetS has been epidemiologically linked to elevated risk for multiple malignancies through various metabolic mechanisms involving chronic systemic inflammation, insulin resistance, and dysregulated lipid metabolism. Especially in HNC, recent studies demonstrated that MetS and metabolic imbalance conditions may contribute to carcinogenesis, disease progression, and clinical outcomes, but the exact mechanisms behind the association between excess fat accumulation and HNC risk remain unclear. Considering previous studies, pharmacological agents targeting metabolic pathways, including biguanides (metformin), thiazolidinediones, sodium‐glucose cotransporter‐2 (SGLT‐2) inhibitors, and HMG‐CoA reductase inhibitors (statins) are being investigated for potential repurposing in cancer prevention and adjuvant therapy.

**Conclusions:**

Here, we summarize the latest evidence on the relationship between MetS and HNC, highlighting the therapeutic potential of anti‐diabetes drugs and anti‐dyslipidemia drugs in ameliorating various pathological problems in HNC patients with MetS.

## Graphical abstract

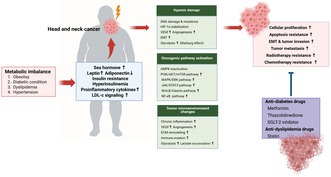

**Abbreviations**
4EBP1: eukaryotic translation initiation factor 4E‐binding protein 1
AdipoR1 & AdipoR2: adiponectin receptor 1 & 2
AGE: advanced glycation end
AKT: protein kinase B
ALDH: aldehyde dehydrogenase
AMP: adenosine monophosphate
AMPK: AMP‐activated protein kinase
APC: adenomatous polyposis coli
ATP: adenosine triphosphate
bFGF: basic fibroblast growth factor
BMI: body mass index
cAMP: cyclic adenosine 3′,5′‐monophosphate
CCL2: CC chemokine ligand 2
CDK: cyclin‐dependent kinase
CI: confidence interval
Cox‐2: cyclooxygenase‐2
CREB: cAMP‐response element binding protein
CSC: cancer stem cell
CVD: cardiovascular disease
DBP: diastolic blood pressure
DDP‐4: dipeptidyl peptidase 4
DM: diabetes mellitus
EGFR: epidermal growth factor receptor
EMT: epithelial‐mesenchymal transition
EREs: estrogen response elements
ERK: extracellular signal‐regulated kinase
ER‐α & ER‐β: estrogen receptors α & β
FPP: farnesyl pyrophosphate
GGPP: geranylgeranyl pyrophosphate
GLUT: glucose transporter
GSH/GPX4: glutathione/glutathione peroxidase
GSK‐3β: glycogen synthase kinase‐3β
HbA1c: glycated hemoglobin
HCC: hepatocellular carcinoma
HDL‐c: high‐density lipoprotein cholesterol
HGF: hepatocyte growth factor
HIF‐1α: hypoxia‐inducible factor‐1 alpha
HMG‐CoA: 3‐hydroxy‐3‐methylglutaryl coenzyme A
HNC: head and neck cancers
HNSCC: head and neck squamous cell carcinoma
HPV: human papillomavirus
HR: hazard ratio
IGF‐1: insulin like growth factor‐1
IGF‐1R: insulin like growth factor‐1 receptor
IGFBPs: IGF‐binding proteins
IL‐6: interleukin‐6
IL‐8: interleukin‐8
IRS1 & IRS2: insulin receptor substrate 1&2
JAK2: janus kinase 2
LDL: low‐density lipoprotein cholesterol
LDLR: LDL receptor
MAPK: mitogen‐activated protein kinase
MCP‐1: monocyte chemoattractant protein‐1
MDR: multidrug resistance
MEK: MAPK/ERK kinase
MetS: metabolic syndrome
MMPs: matrix metalloproteinases
m‐TOR: mammalian target of rapamycin
NADPH: nicotinamide adenine dinucleotide phosphate
NF‐κB: nuclear factor‐kappa B
NPC: nasopharyngeal carcinoma
NSCLC: non‐small cell lung cancer
OB‐R: leptin receptor
OR: odds ratio
PI3K: phosphoinositide 3‐kinase
PIP3: phosphatidylinositol (3,4,5)‐trisphosphate
PPAR‐α: peroxisome proliferator‐activated receptor alpha
PPARγ: peroxisome proliferator‐activated receptor gamma
PTEN: phosphatase and tensin homolog deleted on chromosome 10
RAAS: renin‐angiotensin system
RAC: ras‐related C3 botulinum toxin substrate
RAF: rapidly accelerated fibrosarcoma
RAGE: age receptors
RAP: ras‐related protein
RAS: rat sarcoma
RHO: ras homologous
RNS: reactive nitrogen species
ROS: reactive oxygen species
RR: relative risk
S6K: S6 kinase
SBP: systolic blood pressure
SGLT‐2: sodium‐glucose cotransporter‐2
SHP2: src homology 2‐containing protein tyrosine phosphatase 2
SREBPs: sterol regulatory element‐binding proteins
STAT3: signal transducer and activator of transcription 3
T2D: type 2 diabetes
TAMs: tumor‐associated macrophages e 4
TME: tumor microenvironment
TNF‐α: tumor necrosis factor‐alpha
TSC2: tuberous sclerosis complex 2
TZD: thiazolidinedione
VEGF: vascular endothelial growth factor
WHO: World Health Organization
XIAP: X‐linked inhibitor of apoptosis protein

## 1 Introduction

Head and neck cancer (HNC) encompasses a group of malignancies arising in the oral cavity, pharynx, nasopharynx, larynx, sinuses, and salivary glands [1, 2]. Globally, HNC ranks as the seventh most frequently diagnosed cancer with an estimated annual incidence of 946,456 new cases and 482,001 associated mortalities in 2022 [3, 4]. Among these malignancies, head and neck squamous cell carcinoma (HNSCC) constitutes over 90% of cases. The incidence of HNSCC is projected to increase by 30%, underscoring its escalating public health importance [4, 5, 6]. Metabolic syndrome (MetS) is a collection of interrelated conditions, including hypertension, elevated blood glucose levels, central obesity, and abnormal lipid profiles, that collectively heighten the risk of cardiovascular disease (CVD) and type 2 diabetes (T2D) [7]. The global prevalence of MetS is increasing, affecting over 25% of adults worldwide [8]. MetS is increasingly recognized as a precursor to cancer. Numerous studies have identified a strong association between MetS and its components with an elevated risk of cancer development and mortality. MetS has been implicated as a contributing factor in the increased incidence of liver, colorectal, pancreatic, endometrial, and breast cancers, likely due to its role in promoting systemic inflammation, insulin resistance, and metabolic disturbances that support tumor growth [8, 9, 10, 11, 12, 13, 14, 15, 16, 17, 18]. Recent studies have also demonstrated that MetS or components of MetS contribute to an increased risk of HNC, with evidence showing its association with tumor development, progression, and poor prognosis in affected patients [8, 19, 20].

Given these studies on the relationship between MetS and cancer, researchers are now investigating whether anti‐metabolic syndrome drugs may also play a role in the treatment and prevention of cancer. Some studies have demonstrated that various agents, including metformin, thiazolidinediones, statins, sodium‐glucose cotransporter‐2 (SGLT‐2) inhibitors, and fibrates, have anticancer effects through mechanisms such as AMP‐activated protein kinase (AMPK) activation, mammalian target of rapamycin (mTOR) inhibition, modulation of lipid metabolism, and inhibition of inflammation [21, 22]. In this review, we summarize recent studies to assess whether anti‐metabolic syndrome drugs have a potential therapeutic role in the prevention and treatment of HNC.

## 2 Association Between Cancer and Metabolic Syndrome

### 2.1 Definition of Metabolic Syndrome

According to the International Diabetes Federation (IDF) Task Force on Epidemiology and Prevention, MetS comprises abdominal obesity (defined as a waist circumference of ≥ 90 cm in men and ≥ 80 cm in women for Asians) and at least two of the following components: elevated blood pressure (systolic ≥ 130 and/or diastolic ≥ 85 mmHg), hyperglycemia (fasting plasma glucose ≥ 5.6 mmol/L [equivalent to ≥ 100 mg/dL]), hypertriglyceridemia (triglycerides ≥ 150 mg/dL), and low high‐density lipoprotein cholesterol (HDL‐c) levels (< 40 mg/dL in men and < 50 mg/dL in women). Body mass index (BMI) was calculated as weight in kilograms divided by the square of height in meters (kg/m^2^) [23, 24]. MetS is a significant public health concern due to its strong association with cardiovascular morbidity and T2D [7]. MetS, which includes obesity, hyperinsulinemia, insulin resistance, chronic inflammation, hyperglycemia, and dyslipidemia, contributes to tumor development through a variety of mechanisms.

### 2.2 Cancers Associated With Metabolic Syndrome

MetS has been identified as a significant risk factor for several types of cancer. Research has shown that metabolic syndrome is strongly associated with an elevated risk of pancreatic cancer, colorectal cancer, breast cancer, prostate cancer, thyroid cancer, and endometrial cancer [8, 9, 10, 11, 12, 13, 14, 15, 16, 17, 18]. MetS has been widely studied as a significant risk factor for hepatocellular carcinoma (HCC). The systematic review and meta‐analysis found that MetS increases the risk of HCC by 43% in men (Relative Risk [RR]: 1.43, 95% confidence interval [CI]: 1.19–1.72, *p* < 0.0001) [8]. The study found that MetS is a significant risk factor for HCC, with individuals having MetS showing a 2.13‐fold increased risk of developing HCC compared to those without it (HR: 2.13; 95% CI: 1.96–2.31; *p* < 0.001) [11]. Additionally, the risk of HCC was over six times higher (Odds ratio [OR]: 6.45, 95% CI: 2.35–17.75, *p* < 0.001) in individuals with two or more metabolic syndrome components who were not chronically infected with hepatitis B or C viruses [13]. Several epidemiological studies have linked metabolic syndrome to endometrial cancer, primarily due to obesity‐related hormonal imbalances and chronic inflammation. A systematic review and meta‐analysis found that MetS increases the risk of endometrial cancer by 61% (RR: 1.61, 95% CI: 1.20–2.16, *p* = 0.001) in women [8]. Insulin resistance, a key feature of MetS, leads to increased circulating insulin levels, which promote endometrial cell proliferation [25, 26, 27]. Pancreatic cancer has been increasingly linked to metabolic syndrome, with studies highlighting obesity, diabetes, and dyslipidemia as key contributors. A large prospective cohort study found that individuals with MetS had a 31% higher risk of developing pancreatic cancer [28]. Chronic inflammation associated with MetS has been suggested as a key mechanism promoting pancreatic carcinogenesis [29]. Metabolic syndrome is associated with an increased risk of pancreatic cancer, with a significant association in women (RR: 1.58, 95% CI: 1.26–1.99, *p* < 0.0001) [8]. Moreover, a Mendelian randomization study confirmed that insulin resistance contributes to pancreatic cancer risk [30]. Given these previous findings, the various metabolic imbalances seen in patients with metabolic syndrome increase the risk of developing a variety of cancers.

### 2.3 Obesity

The World Health Organization (WHO) characterizes obesity as an excessive or abnormal accumulation of fat that can negatively impact health [31]. This condition arises from a complex interaction of genetic, environmental, behavioral, and socioeconomic influences [32, 33]. To estimate excess body fat, BMI is commonly used as a proxy measurement. Obesity is generally identified when an individual's BMI reaches or exceeds 30 kg/m^2^. Overweight is defined as a BMI between 25.0 and 29.9 kg/m^2^, normal weight as a BMI between 18.5 and 24.9 kg/m^2^, and underweight as a BMI below 18.5 kg/m^2^ [34]. Additionally, obesity can be assessed using the waist‐to‐hip ratio, which is considered elevated when it exceeds 0.90 in men and 0.85 in women. Research indicates that the waist‐to‐hip ratio is a superior predictor of cardiometabolic risk compared to BMI or waist circumference. Moreover, both waist circumference and waist‐to‐hip ratio are comparable to BMI but serve as stronger indicators of cancer risk [35]. Obesity or high BMI has been found to be closely related to the risk of a number of cancers [18, 34]. Numerous studies have revealed that obesity promotes cancer development through chronic inflammation, insulin resistance, and hormonal imbalances, creating a pro‐tumorigenic environment. Additionally, research has shown that altered levels of adipokines and excessive fat deposition contribute to cancer progression by affecting cell proliferation, angiogenesis, and immune responses [34].

#### 2.3.1 Sex Hormones

Sex hormones, particularly estrogen, play a key role in hormone‐dependent cancers like breast and endometrial cancer. In obesity, increased adipose tissue enhances aromatase activity, converting androgens into estrogens and elevating estrogen levels, especially in postmenopausal women. Estrogen regulates cancer proliferation through both genomic and non‐genomic mechanisms. In the genomic pathway, estrogen binds to nuclear estrogen receptors (ER‐α, ER‐β), leading to receptor dimerization and translocation into the nucleus. The activated receptors bind to estrogen response elements (EREs) in the promoter regions of target genes and lead to the increased expression of genes regulating cell cycle progression, such as cyclin D1 and c‐Myc, as well as anti‐apoptotic factors like Bcl‐2, thereby promoting sustained tumor growth. The genomic pathway is particularly significant in hormone receptor‐positive breast and endometrial cancers, where estrogen‐driven transcriptional activation enhances malignant cell proliferation [36, 37, 38]. The non‐genomic pathway operates independently of direct gene transcription and involves the rapid activation of intracellular kinase signaling cascades. Estrogen interacts with membrane‐associated estrogen receptors or G‐protein coupled estrogen receptor (GPER, also known as GPR30) [39], initiating cross‐talk with receptor tyrosine kinases such as epidermal growth factor receptor (EGFR) and insulin‐like growth factor‐1 receptor (IGF‐1R). This leads to the activation of key oncogenic pathways, including the mitogen‐activated protein kinase/extracellular signal‐regulated kinase (MAPK/ERK), the phosphoinositide 3‐kinase/protein kinase B (PI3K/AKT), and Janus kinase 2/signal transducer and activator of transcription 3 (JAK/STAT3), which promote cell survival, proliferation, and resistance to apoptosis. These rapid signaling events contribute to endocrine therapy resistance and enhance the metastatic potential of cancer cells [40, 41]. The interplay between genomic and non‐genomic pathways underscores the complexity of estrogen‐driven tumorigenesis. Targeting both pathways is crucial in the treatment of hormone‐dependent cancers. Endocrine therapies such as selective estrogen receptor modulators like tamoxifen and aromatase inhibitors like letrozole effectively inhibit genomic signaling. However, the activation of non‐genomic pathways often leads to resistance, necessitating the use of PI3K/AKT and MAPK/ERK inhibitors to suppress estrogen‐induced oncogenic signaling. Given that obesity leads to altered secretion of the sex hormone estrogen, which exacerbates cancer development, strategies aimed at weight loss and metabolic control may further mitigate cancer risk and improve cancer treatment outcomes.

#### 2.3.2 Leptin

Leptin, primarily secreted by white adipose tissue, plays a vital role in energy regulation and metabolism [42]. Leptin signaling is primarily mediated through the leptin receptor (OB‐R), activating multiple intracellular pathways that regulate various physiological functions [43]. Recent studies have identified leptin as a key modulator in cancer progression by promoting proliferation, angiogenesis, invasion, and metastasis, as well as by modulating immune responses and inflammation. However, its influence extends beyond metabolic functions, as research increasingly links leptin to cancer progression. Obese individuals often exhibit high leptin levels, leading to leptin resistance, which has been implicated in tumor development. Leptin enhances insulin sensitivity and regulates lipid metabolism by promoting fatty acid oxidation and suppressing lipogenesis, thereby reducing lipid accumulation in the liver and muscles. These metabolic effects suggest a potential connection between leptin dysregulation and cancer [42, 44, 45, 46]. Leptin mediates its biological effects through its interaction with the long isoform of the leptin receptor (OB‐Rb), leading to the activation of multiple downstream signaling pathways that play critical roles in carcinogenesis. One of the primary signaling mechanisms activated by leptin is the JAK2/STAT3 pathway. Upon leptin binding, JAK2 is phosphorylated, leading to the recruitment and phosphorylation of STAT3, which then translocates to the nucleus and regulates the expression of genes involved in cell survival, proliferation, and inflammation. This pathway is known to upregulate the expression of oncogenes such as c‐Myc and Bcl‐2 while enhancing the production of inflammatory cytokines like interleukin‐6 (IL‐6) and tumor necrosis factor‐alpha (TNF‐α), thereby creating a tumor‐promoting microenvironment [47, 48, 49, 50, 51, 52, 53]. Another key pathway influenced by leptin is the PI3K/AKT signaling cascade. Leptin activates the PI3K signaling pathway by binding to its receptor OB‐Rb, leading to the phosphorylation of JAK2. This activation recruits Insulin receptor substrate 2 (IRS2), which subsequently stimulates PI3K to generate Phosphatidylinositol (3,4,5)‐trisphosphate (PIP3). PIP3 then facilitates the activation of AKT, which promotes cell proliferation, survival, and anti‐apoptotic signaling. This activation enhances cell survival by upregulating anti‐apoptotic proteins such as Bcl‐xL and X‐linked inhibitor of apoptosis protein (XIAP). Moreover, PI3K/AKT signaling plays a crucial role in metabolic reprogramming, enabling cancer cells to meet their energy demands for uncontrolled proliferation [49, 50, 54, 55, 56, 57].

Additionally, leptin activates the MAPK/ERK signaling cascade, which is involved in cellular proliferation and differentiation. Leptin binds to OB‐Rb, activating JAK2, which subsequently recruits SHP2 (Src homology 2‐containing protein tyrosine phosphatase 2). SHP2 activates RAS (Rat sarcoma virus), which then triggers the RAF (Rapidly Accelerated Fibrosarcoma)‐MEK (MAPK/ERK kinase)—ERK cascade, leading to ERK1/2 phosphorylation. This cascade ultimately enhances the transcription of genes associated with cell cycle progression, such as cyclin D1, c‐Myc, c‐Fos, c‐Jun, Elk‐1, and cAMP‐response element binding protein (CREB), thereby contributing to increased tumor cell proliferation [50, 57, 58, 59, 60, 61]. Leptin's role in angiogenesis is largely mediated by its ability to stabilize hypoxia‐inducible factor‐1 alpha (HIF‐1α), which in turn enhances the transcription of vascular endothelial growth factor (VEGF). This process facilitates the formation of new blood vessels, ensuring an adequate supply of nutrients and oxygen to rapidly growing tumor cells. Given these signaling cascades, leptin plays a central role in fostering an environment conducive to cancer development and progression [18, 62]. High leptin levels in obese patients increase the risk of developing cancer and increase the risk of cancer progression.

#### 2.3.3 Adiponectin

Adiponectin, an adipokine primarily secreted by adipose tissue, plays a crucial role in metabolic regulation, insulin sensitivity, and inflammation [18, 63]. Epidemiological studies have demonstrated an inverse relationship between adiponectin levels and various cancers, such as breast, colorectal, and prostate cancer. Lower levels of adiponectin are frequently observed in obesity, a condition that is strongly linked to increased cancer risk and progression [64, 65]. The tumor‐suppressive effects of adiponectin are largely mediated through its receptors, adiponectin receptor 1 and adiponectin receptor 2 (AdipoR1 and AdipoR2), which activate key pathways, including AMPK and Peroxisome proliferator‐activated receptor alpha (PPAR‐α). These pathways inhibit tumor growth by reducing cellular proliferation and promoting apoptosis. Additionally, adiponectin downregulates PI3K/AKT and mTOR signaling, which are critical for cancer cell survival and metabolism [45, 51]. Adiponectin also exerts anti‐inflammatory and anti‐angiogenic effects, which contribute to its protective role against cancer. It suppresses nuclear factor‐kappa B (NF‐κB) signaling, thereby reducing inflammation and cytokine production, both of which are key contributors to the tumor microenvironment [66, 67]. Furthermore, adiponectin decreases the expression of VEGF, thereby inhibiting angiogenesis and limiting tumor growth and metastasis [68, 69]. Given its positive role in cancer prevention and progression, adiponectin is emerging as a potential biomarker for cancer prognosis and therapeutic targets. Strategies to increase adiponectin levels in obese patients by weight loss, physical activity, and pharmacologic interventions may represent a novel approach to cancer prevention and treatment.

#### 2.3.4 Proinflammatory Cytokines

Obesity induces chronic low‐grade inflammation, primarily driven by the secretion of pro‐inflammatory cytokines such as TNF‐α, IL‐6, interleukin‐8 (IL‐8), and monocyte chemoattractant protein‐1 (MCP‐1) from adipocytes and infiltrating macrophages. Hypoxia in expanding adipose tissue exacerbates cytokine production, triggering angiogenesis and insulin resistance. TNF‐α and IL‐6 activate NF‐κB and MAPK pathways, leading to increased reactive oxygen species (ROS), which further promote inflammation and metabolic dysfunction. This inflammatory environment contributes to tumor progression by enhancing cell proliferation, survival, and immune evasion [9, 18, 70]. Inflammatory cytokines play a direct role in cancer development by influencing cell cycle regulation, apoptosis, and oncogene expression. IL‐6, significantly elevated in obese individuals, has been linked to breast, prostate, and hematologic malignancies, with particularly high levels observed in hormone‐resistant tumors. TNF‐α, while capable of inducing apoptosis via mTOR inhibition, paradoxically enhances cell survival and proliferation through NF‐κB and MAPK activation, creating a tumor‐promoting inflammatory state [71]. Moreover, obesity‐related inflammation disrupts insulin signaling, further increasing cancer risk. TNF‐α induces the phosphorylation of IRS‐1 and IRS‐2, interfering with insulin receptor tyrosine kinase activity and contributing to insulin resistance. This metabolic disruption leads to hyperinsulinemia and increased insulin‐like growth factor‐1 (IGF‐1) signaling, both of which drive tumor growth. Given these strong links between inflammation, insulin resistance, and cancer, targeting inflammatory pathways and metabolic dysregulation may be highly effective in reducing obesity‐related cancer risk.

### 2.4 Diabetes

T2D is increasingly recognized as a major factor influencing cancer development and progression. According to the WHO, T2D is a chronic metabolic disorder characterized by the body's inability to effectively utilize insulin, primarily resulting from excess body weight and physical inactivity. The diagnosis of T2D is based on the presence of clinical symptoms such as polyuria or polydipsia, along with at least one of the following criteria: (1) random blood plasma glucose concentration ≥ 11.1 mmol/L, (2) fasting plasma glucose concentration ≥ 7.0 mmol/L (or whole blood ≥ 6.1 mmol/L), (3) 2‐h plasma glucose concentration ≥ 11.1 mmol/L following a 75 g oral glucose tolerance test, or (4) glycated hemoglobin (HbA1c) level of 6.5% or higher (≥ 48 mmol/mol) [31, 72, 73]. The hyperglycemia and insulin resistance/hyperinsulinemia that accompany diabetes play an important role in promoting tumorigenesis through a variety of metabolic, inflammatory, and molecular mechanisms [21, 22, 31, 74].

#### 2.4.1 Hyperglycemia

Hyperglycemia, a key feature of metabolic syndrome, plays a significant role in cancer development and progression by providing cancer cells with an abundant energy source [74, 75]. Cancer cells exhibit an increased metabolic rate and a high demand for glucose, which they meet through the overexpression of glucose transporters, such as glucose transporter (GLUT) 1, GLUT3, and GLUT12, in various tumors. This enhanced glucose uptake fuels glycolysis and adenosine triphosphate (ATP) production, supporting rapid proliferation and survival. Studies have shown that tumors with increased glucose uptake are often associated with higher grades, greater metastatic potential, reduced therapy response, and poorer survival. Furthermore, caloric restriction has been demonstrated to inhibit cancer progression in animal models, suggesting that excess energy availability favors tumor growth [76, 77, 78]. Hyperglycemia also drives oncogenic signaling pathways, further promoting tumorigenesis. High glucose levels activate PI3K/AKT/mTOR signaling, a critical pathway in cancer cell survival and proliferation. Additionally, excess glucose enhances insulin and IGF‐1 signaling, both of which promote cell cycle progression and inhibit apoptosis. The Warburg effect, in which cancer cells rely on glycolysis even in the presence of oxygen, is further amplified under hyperglycemic conditions, creating a favorable environment for tumorigenesis. Moreover, glucose metabolites, such as lactate, contribute to tumor microenvironment acidification, which enhances angiogenesis, immune evasion, and metastasis [79, 80, 81]. Another key mechanism linking hyperglycemia to cancer is the excess production of ROS. High glucose levels promote oxidative stress, leading to DNA damage, mutations in oncogenes such as RAS and tumor suppressor genes such as p53, and chromosomal instability. ROS are generated through multiple pathways, including the polyol/sorbitol pathway, which depletes nicotinamide adenine dinucleotide phosphate (NADPH) and weakens antioxidant defenses, and advanced glycation end product (AGE) formation, which binds to AGE receptors (RAGE) on immune and endothelial cells, further increasing ROS production. This oxidative stress‐induced DNA damage is a major driver of cancer initiation and progression [79, 82].

Given the strong link between hyperglycemia, oxidative stress, and oncogenesis, targeting hyperglycemia may offer promising therapeutic and preventive strategies against cancer. Chronic hyperglycemia contributes to tumor development by promoting glucose‐driven proliferation, activating oncogenic signaling pathways, enhancing oxidative stress, and altering the tumor microenvironment. Therefore, effective management of blood glucose levels through metabolic control—particularly in individuals with metabolic syndrome or diabetes—may play a crucial role in reducing cancer risk and improving clinical outcomes [76].

#### 2.4.2 Insulin Resistance and Hyperinsulinemia

Insulin resistance promotes cancer proliferation through increased bioavailability of IGF‐1. In insulin‐resistant states, elevated insulin levels suppress IGF‐binding proteins (IGFBPs), leading to higher circulating free IGF‐1. IGF‐1 then binds to its IGF‐1R, activating key oncogenic pathways such as PI3K/AKT/mTOR and MAPK/ERK, which drive cell proliferation, survival, and metabolic adaptation in cancer cells. Additionally, IGF‐1R signaling inhibits apoptosis and enhances angiogenesis, creating a tumor‐promoting microenvironment. These mechanisms explain why insulin resistance is strongly correlated with increased cancer risk and poor prognosis in metabolic disorders [83]. Chronic IGF‐1 signaling exacerbates tumor progression by supporting cancer cell metabolism and therapy resistance. The PI3K/AKT pathway, a key mediator of insulin and IGF‐1 signaling, promotes glycolysis and lipid biosynthesis, which are crucial for rapidly dividing cancer cells. Furthermore, sustained IGF‐1R activation induces the epithelial‐mesenchymal transition (EMT), increasing cancer invasiveness and metastasis. Targeting IGF‐1/IGF‐1R signaling has been explored as a therapeutic strategy, with IGF‐1R inhibitors showing potential in insulin‐resistant cancers, particularly in breast, colorectal, and prostate cancer [22, 84].

Given these associations, therapeutic strategies targeting hyperglycemia and insulin resistance may play a crucial role in cancer prevention and treatment in patients with diabetes. Caloric restriction and intermittent fasting have demonstrated benefits in improving insulin sensitivity and reducing tumor growth. Pharmacological interventions such as metformin, which lowers blood glucose levels and improves insulin sensitivity, have shown promise in reducing cancer incidence and improving treatment outcomes. Dietary modifications, including increased consumption of antioxidant‐rich foods containing polyphenols, omega‐3 fatty acids, and selenium, may help counteract oxidative stress and inflammation. Collectively, controlling blood glucose levels and improving insulin sensitivity may serve as effective strategies in reducing cancer risk and improving patient outcomes [85].

### 2.5 Dyslipidemia

Dyslipidemia was defined as serum levels of total cholesterol, low‐density lipoprotein cholesterol (LDL‐C), triglycerides, apolipoprotein B, or lipoprotein(a) exceeding the 90th percentile, or levels of HDL‐C or apolipoprotein falling below the 10th percentile relative to the general population [31]. Dyslipidemia is associated with alterations in lipid metabolic pathways that contribute to cancer progression, metastasis, and drug resistance. Cancer cells exhibit an increased demand for lipids, which serve as an energy source and building blocks for rapidly dividing cells. Dysregulation of fatty acid synthesis and cholesterol metabolism provides cancer cells with the necessary components to sustain their growth and evade apoptosis. Chronic inflammation is a well‐documented consequence of dyslipidemia and a key driver of oncogenesis. Hyperlipidemia is associated with elevated levels of pro‐inflammatory cytokines, oxidative stress, and immune system dysregulation, creating a tumor‐promoting microenvironment. Lipid‐rich tumor microenvironments have been shown to influence immune cell function, potentially leading to immune evasion by cancer cells [9, 18, 86]. LDL‐c plays a crucial role in tumor development and progression by activating multiple oncogenic signaling pathways that enhance cell proliferation, survival, and metastasis. LDL‐c promotes cancer progression by binding to the LDL receptor (LDLR), leading to cholesterol uptake by tumor cells. This process activates the PI3K/AKT/mTOR signaling pathway, which induces the transcription of sterol regulatory element‐binding proteins (SREBPs). These transcription factors enhance cholesterol synthesis and uptake, creating a lipid‐rich microenvironment that supports tumor growth and metastasis [87]. Elevated LDL‐c levels are linked to colorectal cancer metastasis through the activation of the MAPK pathway. LDL‐c enhances ROS production, which in turn upregulates genes involved in cell migration and invasion. This process facilitates tumor progression and resistance to apoptosis [88]. In breast cancer, LDL‐c has been shown to activate the ERK pathway, driving EMT. This transition is characterized by the loss of adhesion molecules such as claudin‐7 and E‐cadherin, leading to increased tumor invasiveness and metastasis [89]. In HCC, LDL receptor inhibition has been associated with increased intracellular cholesterol synthesis through activation of the MEK/ERK pathway. This increase in cholesterol availability promotes cancer cell proliferation and metastasis, demonstrating the metabolic dependency of liver tumors on lipid metabolism [90]. LDL‐c has been shown to activate the JAK/STAT3 pathway in endometrial carcinoma and prostate cancer. This pathway enhances tumor cell proliferation, migration, and invasion. Pharmacological inhibition of JAK2 significantly reduces LDL‐induced tumor growth, indicating that LDL‐c functions as a pro‐tumorigenic factor through this signaling axis [91, 92]. Considering this relationship between cancer and dyslipidemia, regular management of the lipid profile is critical to attenuate the risk of cancer.

### 2.6 Hypertension

Hypertension, as defined by the WHO, is characterized by a systolic blood pressure (SBP) of ≥ 140 mmHg or a diastolic blood pressure (DBP) of ≥ 90 mmHg on two separate occasions [31]. Hypertension is a well‐established risk factor for cardiovascular disease, but emerging evidence suggests a potential association with cancer incidence and mortality. Recent large‐scale studies have provided evidence supporting a modest but significant link between hypertension and cancer incidence. A pooled analysis by Harding et al. reported that untreated hypertension was associated with a modest but statistically significant increase in cancer risk (HR: 1.06, 95% CI: 1.00–1.11, *p* = 0.041), while treated hypertension also showed a significant association with cancer development (HR: 1.09, 95% CI: 1.02–1.16, *p* = 0.008) [93]. Another meta‐analysis of 25 studies with 1.95 million participants found that hypertension increases the risk of colorectal cancer by 15% (RR: 1.15, 95% CI: 1.08–1.23, *p* < 0.001) [94]. A meta‐analysis of 30 studies with 11,643 breast cancer cases found that hypertension was associated with a 15% increased risk of breast cancer (RR: 1.15, 95% CI: 1.08–1.22, *p* < 0.001), with a stronger association in postmenopausal women (RR: 1.20, 95% CI: 1.09–1.31, *p* < 0.001), but no significant risk increase in premenopausal women (RR: 0.97, 95% CI: 0.84–1.12, *p* = 0.66) or the Asian population (RR: 1.07, 95% CI: 0.94–1.22, *p* = 0.31) [95]. In addition to its impact on cancer incidence, hypertension has been associated with poorer survival outcomes in cancer patients. Petrelli et al. conducted a systematic review and meta‐analysis, demonstrating that pre‐existing hypertension was linked to higher cancer‐related mortality (HR: 1.28, 95% CI: 1.21–1.36, *p* < 0.001) and increased recurrence rates (HR: 1.29, 95% CI: 1.10–1.52, *p* = 0.002), particularly among patients with uncontrolled blood pressure [96]. Similarly, one study reported that hypertension was significantly associated with increased mortality from colorectal (OR: 1.22, 95% CI: 1.05–1.42, *p* = 0.01), lung (OR: 1.17, 95% CI: 1.04–1.31, *p* = 0.008), and kidney cancers (OR: 1.75, 95% CI: 1.61–1.90, *p* < 0.001) [97].

These findings highlight the need for effective blood pressure management in cancer patients to potentially improve prognosis and reduce treatment‐related complications. While the biological mechanisms underlying this association remain unclear, chronic inflammation, oxidative stress, and endothelial dysfunction have been proposed as potential mediators linking hypertension to carcinogenesis. Hypertension leads to persistent vascular inflammation, which generates excessive ROS and reactive nitrogen species (RNS), causing oxidative/nitrosative stress. These reactive intermediates induce DNA damage, lipid peroxidation, and protein modifications, all of which contribute to genomic instability and tumor initiation [98, 99, 100]. Moreover, inflammatory cells and cytokines released in hypertensive conditions create a self‐sustaining cycle of tissue damage and repair, further promoting angiogenesis, immune evasion, and the progression of preneoplastic lesions into malignant tumors. Key molecular pathways linking hypertension‐induced inflammation to cancer include NF‐κB activation, prostaglandin overproduction via cyclooxygenase −2 (COX‐2), and disruption of tumor suppressor genes such as p53. These alterations enhance oncogenic signaling while impairing apoptosis and DNA repair mechanisms, creating a pro‐carcinogenic microenvironment. The interplay between chronic inflammation, oxidative stress, and metabolic dysfunction in hypertension underscores the need for targeted interventions to mitigate both cardiovascular and cancer risks [100, 101]. Additionally, the renin‐angiotensin system (RAAS) is implicated in both hypertension and cancer progression, as angiotensin II enhances angiogenesis and tumor growth through increased vascular permeability and cell proliferation [102]. Furthermore, alterations in calcium and cyclic adenosine monophosphate (cAMP) signaling pathways have been associated with both hypertension and tumorigenesis, suggesting that dysregulated intracellular signaling could facilitate cancer progression [103].

Despite growing evidence, the causal relationship between hypertension and cancer remains uncertain due to potential confounding factors and methodological limitations in existing studies. Many studies fail to adequately control for lifestyle factors such as obesity, smoking, and diet, which are independently associated with both hypertension and cancer. Future research should focus on long‐term prospective studies with rigorous adjustments for confounders to clarify the role of hypertension in cancer pathogenesis and progression. Understanding these relationships may have important implications for cancer prevention and management, particularly in the context of personalized medicine and risk stratification [9].

## 3 Metabolic Syndrome and Head and Neck Cancer

### 3.1 MetS and HNC

HNC refers to a group of malignancies that originate in the mucosal linings of the upper aerodigestive tract, including the oral cavity, pharynx, larynx, and nasal passages. These cancers are primarily squamous cell carcinomas, accounting for over 90% of cases. The major risk factors for HNC include tobacco use, excessive alcohol consumption, and persistent human papillomavirus (HPV) infection, particularly HPV‐16 and HPV‐18. Tobacco and alcohol have a synergistic effect, greatly increasing the risk of developing HNSCC. HPV‐associated oropharyngeal cancer has emerged as a distinct clinical entity with a better prognosis compared to non‐HPV‐related HNC. Other notable risk factors include betel quid chewing, poor oral hygiene, chronic irritation, occupational exposure to carcinogens such as wood dust, asbestos, and genetic predisposition [1, 2, 4, 5, 6, 104].

**FIGURE 1:**
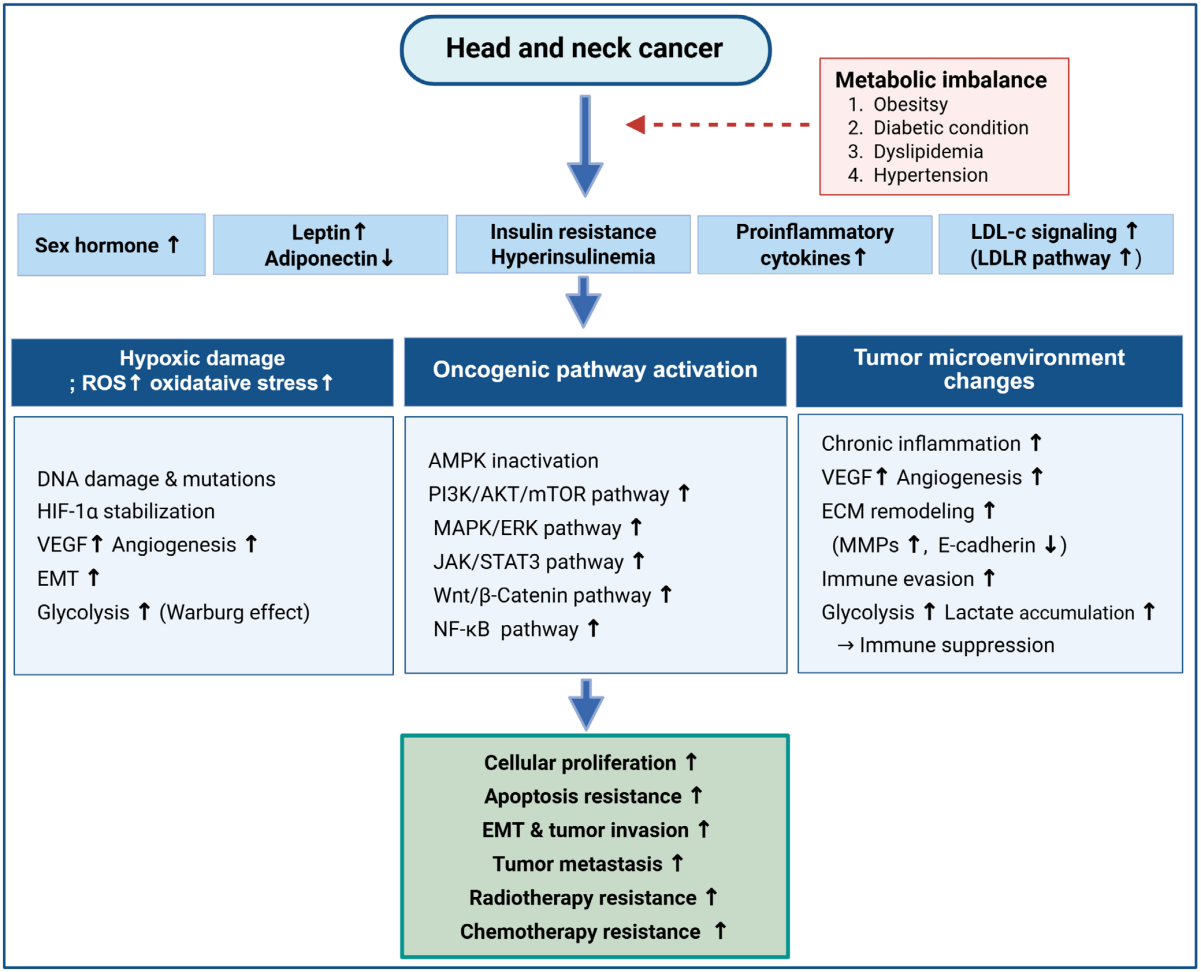
Mechanistic overview of how metabolic imbalance promotes tumor progression in head and neck cancer. Head and neck cancer is affected by metabolic imbalance condition. Increased level of sex hormones, leptin, pro‐inflammatory cytokines, LDL‐c leads to hypoxia condition, oncogenic pathway activation, and tumor microenvironmental changes. These changes boost cellular proliferation, apoptosis resistance, tumor invation, tumor metastasis, radiotherapy resistance and chemotherapy resistance in head and neck cancer. This figure was created with BioRender.com. AKT, protein kinase B; AMPK, AMP‐activated protein kinase; ECM, extracellular matrix; EMT, epithelial‐mesenchymal transition; ERK, extracellular signal‐regulated kinase; HIF‐1α, hypoxia‐inducible factor‐1 alpha; JAK2, janus kinase 2; LDL‐c, low‐density lipoprotein cholesterol; LDLR, LDL receptor; MAPK, mitogen‐activated protein kinase; MMP, matrix metalloproteinase; mTOR, mammalian target of rapamycin; PI3K, phosphoinositide 3‐kinase; ROS, reactive oxygen species; STAT3, signal transducer and activator of transcription 3; VEGF, vascular endothelial growth factor.

As the association between MetS and cancer has been established, research is increasingly focusing on the potential link between MetS and HNC (Figure 1). One study reported MetS was associated with a 1.06‐fold increased risk of HNC (HR: 1.06, 95% CI: 1.01–1.10, *p* < 0.05) in a large 10‐year cohort study. Specifically, the risk was 1.12‐fold higher for oral cavity cancer (HR: 1.12, 95% CI: 1.03–1.23, *p* < 0.05) and 1.18‐fold higher for laryngeal cancer (HR: 1.18, 95% CI: 1.09–1.27, *p* < 0.001) [105]. Another study reported MetS was associated with a 1.13‐fold increased risk of laryngeal cancer (HR: 1.13, 95% CI: 1.07–1.19, *p* < 0.001), with a stronger effect observed in smokers (HR: 3.98, 95% CI: 3.31–4.80, *p* < 0.001) but still significant in non‐smokers (HR: 1.22, 95% CI: 1.01–1.48, *p* = 0.041), indicating MetS as an independent risk factor for laryngeal cancer [106]. While other studies reported MetS itself was not significantly associated with the risk of HNC [19, 20]. Even though the relationship between HNC and MetS is unclear and controversial until now, we need to discuss the possibility of HNC and MetS. The relationship between HNC and MetS remains unclear and controversial, but given the growing number of patients with Mets, the association between HNC and MetS and the potential for combined treatment should be discussed.

### 3.2 Obesity and HNC

Obesity influences cancer development and progression through multiple biological pathways, including chronic inflammation, hormonal dysregulation, and insulin resistance. Chronic inflammation plays a role as adipose tissue secretes pro‐inflammatory cytokines such as TNF‐α and IL‐6, which promote tumor progression and angiogenesis. Hormonal dysregulation is another contributing factor, as obesity is associated with increased leptin, a pro‐tumorigenic hormone, and decreased adiponectin, an anti‐tumorigenic hormone, which alters immune responses and tumor growth dynamics (Figure 1). Insulin resistance and IGF‐1 signaling are also mechanisms by which obesity affects cancer progression since hyperinsulinemia leads to increased IGF‐1, enhancing tumor cell proliferation and metastasis [107, 108] (Figure 1). The relationship between obesity and HNC incidence is complex and inconsistent across studies. Some research indicates an increased risk, while others suggest a protective effect or no significant association. A meta‐analysis of waist circumference and HNC risk reported that obesity, measured by waist circumference, increased HNC risk in men and in women with a waist circumference of at least 93.16 cm [109]. Another study indicated that abdominal obesity is linked to increased HNC risk, supporting the role of central adiposity as a key factor in cancer risk [8]. On the other hand, some studies suggest that obesity reduces HNC risk. A pooled analysis from the International Head and Neck Cancer Epidemiology (INHANCE) Consortium found that underweight individuals with a BMI of 18.5 or less had a significantly higher risk of HNC (OR: 2.13, 95% CI: 1.75–2.58, *p* < 0.001), whereas overweight individuals had a reduced risk (OR: 0.52, 95% CI: 0.44–0.60, *p* < 0.001) and obese individuals showed an even lower risk (OR: 0.43, 95% CI: 0.33–0.57, *p* < 0.001) [110]. Another study found that obesity had a protective effect against HNC in men, where higher BMI was associated with a decreased risk (HR = 0.804, 95% CI: 0.765–0.846, *p* < 0.001) [111]. The impact of obesity on HNC prognosis is similarly debated, with studies showing either improved or worsened survival outcomes or no significant correlation. A 2023 cohort study of HNC patients undergoing curative‐intent definitive chemoradiation found that overweight patients had significantly better overall survival (HR = 0.59, 95% CI: 0.39–0.91, *p* = 0.02) and progression‐free survival (HR = 0.51, 95% CI: 0.34–0.75, *p* < 0.001) compared to normal‐weight patients [112]. Conversely, some studies indicate that obesity is associated with poor prognosis. A study on sarcopenic obesity in HNC patients undergoing radiotherapy found that 28% had this condition and were four times more likely to experience critical weight loss during treatment (OR = 4.1, 95% CI: 1.5–7.1, *p* = 0.002) [113]. Obesity was also linked to poor surgical outcomes, including airway management issues, wound infections, and delayed recovery in HNC patients [114]. Other studies reported that while obesity influences treatment‐related complications, it does not significantly impact overall survival [8].

Obesity is associated with poor prognosis in some cancers, but the impact of obesity on HNC remains unclear. Studies have shown that being underweight may be a greater risk factor for HNC, while obesity is often associated with better treatment tolerance and improved survival outcomes. However, obesity raises additional considerations for surgery and radiation therapy in patients with HNC, requiring a personalized treatment approach. Further research is needed to elucidate the exact mechanisms by which obesity is associated with HNC incidence and prognosis, and to optimize cancer treatment for patients who are both obese and have HNC.

### 3.3 Diabetes and HNC

Diabetes mellitus (DM) contributes to HNC development through chronic hyperglycemia, which fuels tumor growth via the Warburg effect, where cancer cells preferentially utilize glycolysis over oxidative phosphorylation. This leads to increased lactate production, acidification of the tumor microenvironment, and degradation of the extracellular matrix, promoting tumor invasion and metastasis. Insulin resistance and hyperinsulinemia in diabetes further drive cancer progression by activating IR and IGF‐1R pathways, particularly the PI3K/AKT/mTOR cascade, which enhances cell proliferation, survival, and angiogenesis (Figure 1). IGF‐1 also stimulates the RAS/RAF/MAPK and JAK/STAT pathways, preventing apoptosis and promoting tumor metastasis (Figure 1). Chronic inflammation and oxidative stress play a critical role in diabetes‐induced carcinogenesis by increasing pro‐inflammatory cytokines such as IL‐6, TNF‐α, NF‐κB and ROS, which lead to DNA damage, genetic mutations, and immune suppression (Figure 1). This inflammatory microenvironment not only facilitates cancer initiation and progression but also enhances radioresistance in HNC patients, worsening treatment outcomes [115, 116]. Several studies have demonstrated that hyperglycemia is an independent risk factor for HNC. One study found that DM was significantly associated with an increased risk of HNC in both men (HR 1.183, 95% CI: 1.114–1.256, *p* < 0.001) and women (HR 1.190, 95% CI: 1.016–1.393, *p* = 0.029) [20]. Another study reported that individuals with elevated blood glucose levels (≥ 4.70 mmol/L) had an increased risk of HNC (HR 1.10, 95% CI: 1.01–1.19, *p* = 0.029), with hyperglycemic patients exhibiting a significantly higher risk (HR 1.22, 95% CI: 1.02–1.45, *p* = 0.028) [19]. Additionally, high fasting blood glucose was strongly correlated with laryngeal cancer (HR 1.123, 95% CI: 1.064–1.186, *p* < 0.001). These findings suggest that persistently high blood glucose levels may contribute to HNC development through mechanisms such as insulin resistance, chronic inflammation, and oxidative stress [106].

### 3.4 Dyslipidemia and HNC

A systematic review and meta‐analysis investigating metabolic traits, including dyslipidemia, in relation to HNC found that low HDL‐c and high triglyceride levels were significantly associated with an increased risk of HNC. The biological mechanism behind this association is thought to involve chronic inflammation and oxidative stress induced by lipid dysregulation. Low HDL‐c levels are linked to impaired lipid transport and increased inflammation, which can promote carcinogenesis in the head and neck region (Figure 1). Similarly, high triglyceride levels have been associated with pro‐tumorigenic signaling pathways, including insulin resistance and metabolic dysregulation, which may contribute to HNC development. One study reported dyslipidemia components, including high triglycerides (HR 1.092, 95% CI: 1.035–1.153, *p* = 0.001) and low HDL‐c (HR 1.087, 95% CI: 1.025–1.153, *p* = 0.005), were significantly linked to an increased risk of laryngeal cancer [106]. Conversely, the other study showed a U‐shaped association with HNC risk in men, where low HDL‐c (< 1.26 mmol/L) decreased risk (HR 0.88, 95% CI: 0.77–1.00, *p* = 0.048), while high HDL‐c (≥ 1.26 mmol/L) increased risk (HR 1.19, 95% CI: 1.06–1.34, *p* = 0.003), whereas triglycerides (HR 0.95, 95% CI: 0.81–1.10, *p* = 0.48) showed no significant association [19]. Regarding the relationship between dyslipidemia and HNC, we should focus on controlling the lipid profile to attenuate the risk of HNC.

### 3.5 Hypertension and HNC

Hypertension has been implicated in HNC development through multiple biological mechanisms. Chronic inflammation and oxidative stress induced by prolonged hypertension contribute to vascular endothelial dysfunction, increased ROS, and elevated pro‐inflammatory cytokines such as IL‐6, TNF‐α, and NF‐κB, which can lead to DNA damage and tumor progression (Figure 1). Additionally, hypertension‐related vascular remodeling and reduced tissue perfusion create a hypoxic tumor microenvironment, promoting angiogenesis, cancer cell survival, and metastasis. Furthermore, elevated levels of angiotensin II, a key regulator in hypertension, have been shown to stimulate tumor growth through the activation of pro‐inflammatory and proliferative pathways. These mechanisms suggest that hypertension may not only be a comorbid condition but also an independent contributor to HNC development and progression. Several studies have explored the association between hypertension and HNC, yielding mixed findings. One study found that there is an increased risk of HNC in men with hypertension (HR 1.049, 95% CI: 1.001–1.101, *p* = 0.045), but no significant association in women [20]. Another study reported that high blood pressure was significantly associated with an increased risk of laryngeal cancer (HR 1.120, 95% CI: 1.056–1.189, *p* < 0.001) [106]. However, some research has found no significant correlation between hypertension and overall HNC risk (HR 1.00, 95% CI: 0.82–1.20, *p* = 0.994), with both systolic and diastolic blood pressure showing no meaningful association with HNC incidence [19]. Given these conflicting results, further studies are needed to better understand the potential role of hypertension in HNC development and progression.

## 4 Regulation of Anti Diabetic Drug in HNC With MetS Patients

### 4.1 Metformin

**FIGURE 2:**
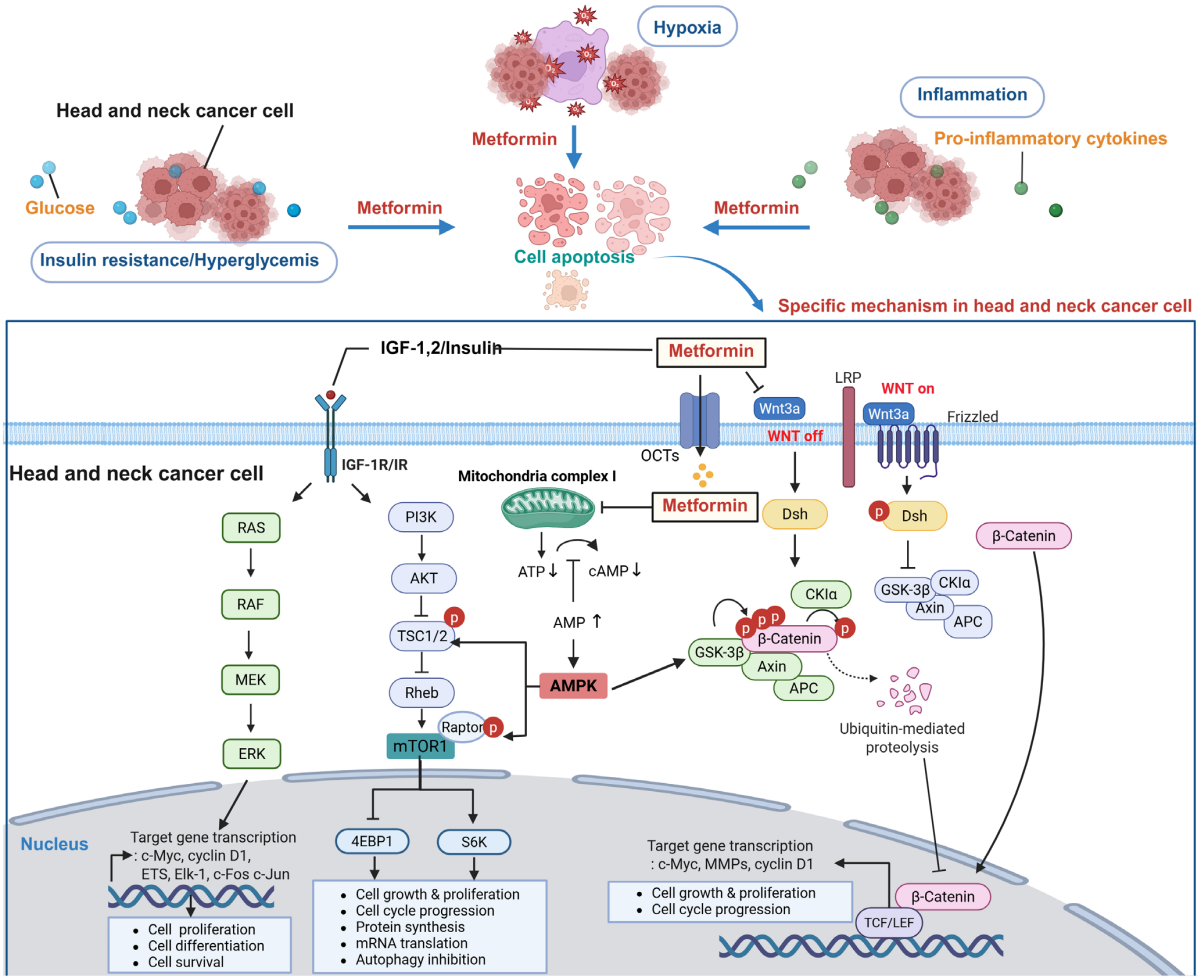
Signaling pathways mediating the anticancer effects of metformin in HNC. Metabolic imbalance including insulin resistance, hyperglycemia, hypoxia and inflammation boosts head and neck cancer survival and proliferation. Metformin contributes to cell apoptosis in head and neck cancer in spite of these metabolic imbalance. Metformin activates AMPK, an energy sensor that inhibits anabolic processes and promotes catabolism, leading to decreased cancer cell growth and proliferation, while suppressing the mTOR pathway via AMPK activation, which reduces protein synthesis and tumor progression. Additionally, metformin downregulates the Wnt/β‐catenin pathway, preventing β‐catenin nuclear accumulation and inhibiting cancer stemness and tumor development. It also lowers IGF‐1R signaling, leading to reduced PI3K/AKT and RAS/RAF/MEK pathway activation, which suppresses survival signals and promotes apoptosis in cancer cells. Through these pathways, metformin exerts broad anticancer effects, including inhibition of proliferation, induction of apoptosis, suppression of metastasis, and reduction of cancer stem cell populations. This figure was created with BioRender.com. 4EBP1, eukaryotic translation initiation factor 4E‐binding protein 1; AKT, protein kinase B; AMPK, AMP‐activated protein kinase; APC, adenomatous polyposis coli; ERK, extracellular signal‐regulated kinase; GSK‐3β, glycogen synthase kinase‐3β; HIF‐1α, hypoxia‐inducible factor‐1 alpha; MEK, MAPK/ERK kinase; mTOR, mammalian target of rapamycin; PI3K, phosphoinositide 3‐kinase; ROS, reactive oxygen species; S6K, S6 kinase; TSC, tuberous sclerosis complex.

Metformin, a widely used biguanide for T2D [117], has garnered significant attention for its anti‐cancer properties. It exerts its effects through direct and indirect mechanisms, targeting key oncogenic signaling pathways that regulate cell proliferation, apoptosis, metabolism, and immune evasion [118, 119] (Figure 2).

#### 4.1.1 AMPK Activation and mTOR Inhibition

The mTOR pathway is a central regulator of cell growth, metabolism, and proliferation. It exists as two distinct complexes: mTORC1 and mTORC2. The mTORC1 complex is highly sensitive to nutrient availability, energy levels, and growth factors, making it a key driver of protein synthesis, cell cycle progression, and autophagy inhibition. Under normal conditions, mTORC1 is activated by upstream signals such as insulin, IGF‐1, and the AKT/PI3K pathway, leading to phosphorylation of S6 kinase (S6K) and eukaryotic translation initiation factor 4E‐binding protein 1 (4EBP1), both of which promote translation of proteins required for cancer cell survival and proliferation. Dysregulation of mTORC1 is frequently observed in cancers, where it supports uncontrolled tumor growth, angiogenesis, and resistance to apoptosis. mTORC2, on the other hand, regulates cytoskeletal organization and cell survival via activation of AKT, further promoting tumorigenesis. Given its central role in oncogenesis, mTOR hyperactivation is a major target for cancer therapy [21, 120].

Metformin exerts its anti‐cancer effects on the mTOR pathway primarily by activating AMPK, a cellular energy sensor that responds to low ATP levels. Metformin induces mild mitochondrial stress, leading to an increase in the AMP (adenosine monophosphate):ATP ratio, which directly activates AMPK. Once activated, AMPK phosphorylates and inhibits the mTORC1 complex through two key mechanisms. First, AMPK directly phosphorylates tuberous sclerosis complex 2 (TSC2), a negative regulator of mTOR, promoting its GTPase‐activating function and ultimately suppressing mTORC1 activity. Second, AMPK can directly phosphorylate Raptor, a core component of mTORC1, leading to its dissociation and inactivation. This suppression results in reduced translation of oncogenic proteins such as cyclin D1 and c‐Myc, preventing tumor cell proliferation. Additionally, metformin's inhibition of insulin/IGF‐1 signaling further disrupts mTORC1 activation, amplifying its anti‐proliferative effects [121, 122, 123, 124] (Figure 2).

#### 4.1.2 Wnt/β‐Catenin Pathway Inhibition

The Wnt/β‐catenin signaling pathway is a crucial regulator of embryonic development, tissue homeostasis, and stem cell renewal. However, dysregulation of Wnt signaling is frequently observed in various cancers, leading to uncontrolled cell proliferation, invasion, and metastasis. Under normal conditions, the pathway is tightly regulated by the destruction complex, which includes glycogen synthase kinase‐3β (GSK‐3β), adenomatous polyposis coli (APC), and Axin. This complex phosphorylates β‐catenin, marking it for proteasomal degradation. In cancer cells, mutations in APC or β‐catenin, or excessive Wnt ligand activation, result in β‐catenin accumulation and nuclear translocation, where it acts as a transcriptional co‐activator of oncogenic genes, such as c‐Myc, cyclin D1, and matrix metalloproteinases (MMPs). This promotes tumor growth, EMT, and chemoresistance, making Wnt/β‐catenin a key target for cancer therapy [125, 126, 127, 128].

Metformin inhibits Wnt/β‐catenin signaling through multiple mechanisms, indirectly suppressing oncogenic activity and tumor progression. First, metformin activates AMPK, which enhances GSK‐3β activity, reinforcing β‐catenin phosphorylation and promoting its proteasomal degradation. This prevents β‐catenin from translocating to the nucleus, thereby suppressing the transcription of oncogenic genes such as c‐Myc, Cyclin D1, and MMPs (Figure 2). However, it is important to note that metformin does not directly target β‐catenin but rather modulates its stability through upstream regulators [129].

Additionally, metformin has been shown to reduce the secretion of Wnt ligands such as Wnt3a, effectively disrupting Wnt signaling at the extracellular level. This inhibition varies across cell types and tumor microenvironments, and in some cases, β‐catenin suppression may be less pronounced. Another crucial mechanism involves metformin‐induced inhibition of AKT, which normally phosphorylates and inhibits GSK‐3β, thereby stabilizing β‐catenin. By suppressing AKT activity, metformin further ensures that GSK‐3β remains active, leading to continuous degradation of β‐catenin. This aligns with the broader impact of metformin on the PI3K/AKT/mTOR signaling axis, which is also critical for cancer cell metabolism and survival [129] (Figure 2).

These combined actions result in decreased tumor growth, inhibition of cancer stem cell renewal, and reduced metastasis potential in colon, breast, lung, and liver cancers. Since Wnt/β‐catenin signaling is essential for cancer stem cell (CSC) maintenance, metformin's ability to suppress this pathway makes it a powerful agent for targeting CSCs, which are responsible for tumor recurrence and resistance to chemotherapy [125, 130, 131, 132, 133, 134, 135]. A study reported that Wnt inhibitor XAV939 enhances the radiosensitivity of human cervical cancer HeLa cells by inhibiting the Wnt/β‐catenin signaling pathway. This combination treatment with XAV939 and carbon‐ion radiation led to increased DNA damage, G2/M cell cycle arrest, and apoptosis, while reducing the expression of Wnt‐related proteins, suggesting its potential as a radiosensitizer for cervical cancer therapy [136]. A study reported that metformin inhibits the growth and proliferation of ovarian CSCs both in vitro and in vivo, reducing CSC markers like aldehyde dehydrogenase + (ALDH+) and impairing tumor sphere formation. It also reported that metformin enhances the efficacy of chemotherapy such as using cisplatin, decreases angiogenesis, and shows potential as a therapeutic strategy for ovarian cancer treatment [137]. These findings suggest that metformin could be integrated into multi‐targeted cancer therapies to improve patient outcomes by inhibiting Wnt‐driven tumor progression.

#### 4.1.3 IGF‐1 and Insulin Signaling Suppression

Metformin exerts anti‐cancer effects by suppressing IGF‐1 and insulin signaling pathways, which are key regulators of cell growth, metabolism, and tumor progression (Figure 2). IGF‐1 and insulin activate PI3K/AKT/mTOR and RAS/MAPK pathways, promoting cell proliferation, survival, and resistance to apoptosis. Metformin primarily reduces hepatic glucose production and lowers circulating insulin levels, thereby decreasing insulin/IGF‐1 receptor activation in cancer cells. Additionally, metformin inhibits IGF‐1R autophosphorylation, reducing downstream PI3K/AKT/mTOR and MAPK pathway signaling, which limits cancer cell proliferation and survival (Figure 2). Beyond systemic effects, metformin directly inhibits IGF‐1 and insulin signaling at the cellular level via AMPK activation. Activated AMPK negatively regulates mTORC1, reducing protein synthesis and cell growth. Metformin also promotes IGFBP expression, which sequesters IGF‐1, preventing it from binding to IGF‐1R and activating downstream signaling. Furthermore, metformin inhibits IRS‐1 phosphorylation, leading to decreased activation of PI3K/AKT and MAPK cascades, which are essential for tumor growth and progression (Figure 2). This dual suppression of IGF‐1 and insulin signaling is a key mechanism by which metformin exerts its anti‐tumorigenic effects across various cancers [138, 139, 140, 141]. Studies investigating the association between metformin and HNC have reported mixed findings. Some studies suggest that metformin has a significant protective effect, while others indicate no meaningful impact on survival or recurrence (Table 1).

**TABLE 1:** Studies investigating the association between HNC and anti‐diabetes and anti‐dyslipidemia drugs.

Author (year)	Drug	Study designs (clinical trial ID)	Results	Reference
Yen et al. (2015)	Metformin	Prospective cohort	Metformin use in diabetic patients was associated with a 34% lower incidence of head and neck cancer compared to non‐users (HR: 0.66, 95% CI: 0.55–0.79, *p* < 0.001). The protective effect was particularly significant for oropharyngeal cancer (HR: 0.36, 95% CI: 0.17–0.74, *p* = 0.006) and nasopharyngeal carcinoma (HR: 0.50, 95% CI: 0.31–0.80, *p* = 0.004).	[142]
Alcusky et al.(2019)	Metformin	Retrospective cohort	Metformin exposure after HNC diagnosis was associated with a reduced all‐cause mortality rate, with one analysis reporting a 22% lower risk of death in the first 2 years (HR: 0.78, 95% CI: 0.65–0.93, *p* = 0.006), while a separate time‐specific analysis showed a non‐significant reduction within 2 years (HR: 0.81, 95% CI: 0.61–1.09, *p* = 0.16) and no benefit beyond 2 years (HR: 1.20, 95% CI: 0.94–1.53, *p* = 0.14); the protective effect was significant in patients aged ≤ 60 years (HR: 0.22, 95% CI: 0.09–0.56, *p* = 0.001), but not in those older than 60.	[143]
Lee et al. (2019)	Metformin	Retrospective cohort s	There was no significant difference in overall survival (HR 1.04, 95% CI 0.72–1.50, *p* = 0.83), recurrence‐free survival (HR 1.04, 95% CI 0.66–1.62, *p* = 0.88), or disease‐specific survival (HR 1.16, 95% CI 0.68–1.98, *p* = 0.58) between metformin users and non‐users.	[144]
Rego et al. (2017)	Metformin	Systematic review of in vitro studies	Metformin demonstrated anti‐tumor effects in HNSCC cell lines by inhibiting cell proliferation (> 50% reduction, dose‐dependent), inducing G0/G1 cell cycle arrest, and promoting apoptosis. Mechanistically, these effects were linked to the activation of AMPK and inhibition of the mTOR pathway, supporting its potential as an anti‐cancer agent in preclinical models.	[120]
Kwon el al. (2015)	Metformin	Retrospective cohort	Metformin use did not significantly affect the recurrence of primary head and neck cancer (*p* > 0.2) or the occurrence of second primary cancers (*p* > 0.2). Although non‐metformin users had lower overall (*p* = 0.017) and cancer‐specific survival (*p* = 0.054) in univariate analysis, this association was not confirmed in multivariate analysis (HR 1.26, 95% CI 0.82–1.93, *p* = 0.284).	[145]
Kemnade et al. (2023)	Metformin	Phase I/II prospective single‐arm clinical trial (NCT02949700)	This Phase I/II trial assessed metformin as a chemo‐radiosensitizer in HNSCC patients, showing good tolerability but no significant survival benefit. While the ≥ 70% compliance group had a trend toward improved survival, it was not statistically significant (PFS: *p* = 0.16, OS: *p* = 0.19), highlighting the need for further randomized trials.	[146]
Gaertner et al. (2024)	Metformin	Retrospective cohort	Metformin use was associated with a significantly higher 5‐year survival rate in patients with head and neck cancer (75.3% vs. 69.8%, *p* < 0.001), along with a reduced risk of death (HR: 0.78, 95% CI: 0.75–0.82, *p* < 0.001; OR: 0.79, 95% CI: 0.75–0.83, *p* < 0.001)	[147]
Curry et al. (2018)	Metformin	Prospective clinical trial (NCT02083692)	Metformin in HPV+ and HPV− HNSCC found a significantly higher increase in apoptosis in HPV−, tobacco‐associated HNSCC (mean 13.7/high power field) compared to HPV+ oropharyngeal squamous cell carcinoma (mean 5.7/high power field, *p* < 0.001). Additionally, metformin treatment led to an increase in CD8+ T cell infiltrate (22.8% vs. 10.7%, *p* = 0.006) and FoxP3+ regulatory T cell infiltrate (9% vs. 5%, *p* = 0.019) in the tumor microenvironment.	[148]
Ebrahimi et al. (2023)	Metformin	In vitro	An in vitro study using HN5 human HNC cell line found that metformin had a dose‐ and time‐dependent cytotoxic effect on cancer cells while sparing normal cells. The combination of metformin and laser irradiation significantly increased cytotoxicity (*p* < 0.05) and further downregulated the mTOR signaling pathway. This suggests a potential synergistic effect between metformin and laser therapy for HNC treatment.	[149]
Veeramachaneni et al. (2021)	Metformin	Preclinical in vivo	A preclinical study using mEERL95 cells, which are mouse‐derived HPV‐positive HNSCC models, found that chronic metformin treatment significantly reduced tumor growth velocity (> 50%, *p* < 0.0001) and increased the CD8^+^/T‐reg ratio, leading to enhanced tumor immune infiltration. These findings suggest that long‐term metformin administration may potentiate immune checkpoint inhibitors by modulating the tumor immune microenvironment.	[150]
Jiao et al. (2022)	Metformin	Meta‐analysis	A meta‐analysis of found that metformin significantly improved OS in HNC patients (HR = 0.87, 95% CI: 0.76–0.99, *p* = 0.04). However, it did not significantly impact DFS or DSS.	[151]
Lin et al. (2014)	Metformin	In vitro	This study found that metformin significantly enhanced the anticancer effect of dasatinib in HNSCC cells by inducing AMPK‐dependent ER stress. In vitro, metformin combined with dasatinib led to greater apoptosis (*p* < 0.05) and EGFR degradation than dasatinib alone. In vivo, metformin‐dasatinib combination therapy significantly reduced tumor growth in xenograft models (*p* < 0.05), suggesting a synergistic effect that could improve treatment outcomes in HNSCC.	[152]
Wu et al. (2019)	Metformin	In vivo and in vitro	This study demonstrated that metformin directly targets carcinoma‐initiating cells in HNSCC, inhibiting tumor progression via mitochondrial complex I inhibition and AMPK/mTOR pathway suppression.	[153]
Zhao et al. (2011)	Metformin	In vitro	Metformin effectively inhibits nasopharyngeal carcinoma cell proliferation by inducing G1 cell cycle arrest, downregulating cyclin D1, and suppressing mTORC1 signaling through AMPK activation.	[154]
Govindarajan et al. (2017)	TZD	Retrospective Cohort	Patients exposed to TZDs had a significantly lower risk of developing head‐and‐neck cancer compared to those not exposed (HR = 0.43, 95% CI: 0.21–0.89, *p* = 0.023). When combined with other oral antidiabetic agents, the risk reduction remained significant (HR = 0.63, 95% CI: 0.42–0.94, *p* = 0.023), but the benefit was not observed when TZDs were used alongside insulin (HR = 1.30, 95% CI: 0.88–1.92, *P* = 0.19).	[155]
Frohlich and Wahl (2015)	TZD	Retrospective Cohort	TZDs demonstrated a chemopreventive effect, reducing HNC by 40% (HR = 0.60, 95% CI: 0.37–0.98, *P* < 0.05) in diabetic patients.	[156]
Yang et al. (2020)	TZD	Retrospective Cohort	Prior pioglitazone use was associated with a slightly increased risk of head and neck cancer (OR = 1.06, 95% CI: 1.02–1.10, *P* < 0.05), with regular use showing a similar trend (OR = 1.05, 95% CI: 1.01–1.10, *p* < 0.05) while irregular use was not significant (OR = 1.10, 95% CI: 0.98–1.25, *p* > 0.05). Among cancer subtypes, oral cavity cancer had a significantly higher risk (OR = 1.11, 95% CI: 1.06–1.15, *p* < 0.05), while no significant associations were found for salivary gland cancer (OR = 0.99, 95% CI: 0.87–1.14, *p* > 0.05) or nasopharyngeal cancer (OR = 0.69, 95% CI: 0.44–1.06, *p* > 0.05).	[157]
Burotto and Szabo (2014)	TZD	Meta‐analysis Retrospective cohort	Meta‐analysis demonstrated TZDs use is associated with a 41%–55% reduction in HNSCC risk (RR = 0.45–0.59, 95% CI: 0.30–0.85, *p* < 0.05). A large population‐based cohort study also found a 15% risk reduction in HNSCC among TZD users (*p* = 0.04). However, these findings are limited to diabetic populations, and their applicability to non‐diabetic individuals remains uncertain.	[158]
Li. et al. (2024)	SGLT‐2 inhhibitor	Retrospective cohort	Use of SGLT‐2 inhibitor was associated with a 59% lower risk of nasopharyngeal carcinoma compared to DPP‐4 inhibitor (HR: 0.41, 95% CI: 0.21–0.81, *p* < 0.01), but there was no significant association with head and neck cancer overall (HR: 1.00, 95% CI: 0.26–3.92, *p* = 0.997) after adjustments	[159]
Lin et at. (2025)	Statin	Retrospective cohort	Post‐diagnosis statin use may improve survival, with one study reporting a 22% reduction in all‐cause mortality (HR = 0.78, 95% CI: 0.67–0.90, *p* = 0.001)	[160]
Filaferro et at. (2024)	Statin	Systemic review	A systematic review found that 75% of HNC‐related studies indicated a protective effect of statins, with one study showing a 28% lower risk of recurrence (OR = 0.72, 95% CI: 0.58–0.89, *p* = 0.003)	[161]
Rhi et at. (2024)	Statin	Nested case–control	Long‐term statin use (more than 2 years) was associated with a 17% reduced risk of developing HNC (OR = 0.83, 95% CI: 0.75–0.91, *p* < 0.001), while short‐term use (≤ 1 year) showed no significant effect	[162]

Abbreviations: AMPK, AMP‐activated protein kinase; CI, confidence interval; DDP‐4, dipeptidyl peptidase 4; DFS, disease‐free survival; DSS, disease‐specific survival; EGFR, epidermal growth factor receptor; HNC, head and neck cancer; HNSCC, head and neck squamous cell carcinoma; HR, hazard ratio; mTOR, mammalian target of rapamycin; OR, odds ratio; OS, overall survival; PFS, progression free survival; RR, risk ratio; SGLT‐2, sodium‐glucose cotransporter‐2; TZD, thiazolidinedione.

Several epidemiological studies have found a significant association between metformin use and improved outcomes in HNC. Yen et al. reported that diabetic patients using metformin had a lower incidence of HNC, with particularly strong protective effects observed for oropharyngeal and nasopharyngeal cancer (HR: 0.58, 95% CI: 0.44–0.76, *p* < 0.001 for oropharyngeal; HR: 0.41, 95% CI: 0.23–0.73, *p* = 0.002 for nasopharyngeal) [142]. Similarly, Gaertner et al. found that metformin users had a higher 5‐year survival rate (75.3% vs. 69.8%) and a lower risk of death compared to non‐users (HR: 0.78, 95% CI: 0.75–0.82, *p* < 0.001; OR: 0.79, 95% CI: 0.75–0.83, *p* < 0.001), suggesting a potential survival benefit of metformin in patients with HNC [147]. A meta‐analysis by Jiao et al. indicated that metformin significantly improved overall survival in HNC patients (HR: 0.87, 95% CI: 0.76–0.99, *p* = 0.037), with stronger effects observed in high‐quality studies (HR: 0.66, 95% CI: 0.49–0.88, *p* < 0.01) [151]. Alcusky et al. reported that metformin exposure after HNC diagnosis was associated with a significantly reduced all‐cause mortality rate within the first 2 years, particularly among patients aged 60 or younger (HR: 0.78, 95% CI: 0.65–0.93, *p* = 0.006) [143].

In a combined preclinical and clinical study, Curry et al. first showed in a mouse model that metformin treatment increased CD8^+^ T‐cell infiltration and FoxP3^+^ regulatory T‐cell infiltration in the tumor microenvironment, using mEERL95 cells, which are mouse‐derived HPV‐positive HNSCC models. Clinically, metformin induced a significantly higher rate of apoptosis in HPV‐negative, tobacco‐associated HNC compared to HPV‐positive oropharyngeal squamous cell carcinoma, suggesting a consistent immunomodulatory and pro‐apoptotic effect across models [148]. Rego et al. found that metformin inhibited cell proliferation in HNSCC cell lines by over 50% in a dose‐dependent manner, induced G0/G1 cell cycle arrest, and promoted apoptosis through AMPK activation and mTOR inhibition [120]. Several in vivo and in vitro studies have provided further support for metformin's potential anticancer effects in HNC. Wu et al. demonstrated that metformin specifically targeted carcinoma‐initiating cells in HNSCC, inhibiting tumor progression via mitochondrial complex I inhibition and suppression of the AMPK/mTOR pathway [153]. Ebrahimi et al. showed that metformin, when combined with laser irradiation, significantly increased cytotoxic effects on HNC cells while further downregulating the mTOR signaling pathway [149]. Veeramachaneni et al. found that chronic metformin treatment significantly reduced tumor growth in an HPV‐associated HNC mouse model and enhanced immune infiltration, potentially potentiating the effects of immune checkpoint inhibitors [150]. Lin et al. reported that metformin enhanced the anticancer effect of dasatinib in HNSCC cells by inducing AMPK‐dependent ER stress, leading to increased apoptosis and EGFR degradation [152]. Zhao et al. found that metformin effectively inhibited nasopharyngeal carcinoma cell proliferation by inducing G1 cell cycle arrest, downregulating cyclin D1, and suppressing mTORC1 signaling through AMPK activation [154].

Conversely, other studies have found no significant impact of metformin on HNC outcomes. Lee et al. reported no significant difference in overall survival, recurrence‐free survival, or disease‐specific survival between metformin users and non‐users [144]. Similarly, Kwon et al. found that metformin use did not significantly affect HNC recurrence or second primary cancer incidence. While non‐metformin users exhibited lower overall and cancer‐specific survival rates in univariate analysis, multivariate analysis did not confirm a statistically significant association [145]. In clinical trials, Kemnade et al. evaluated metformin as a chemo‐radiosensitizer in HNSCC patients and found good tolerability but no significant survival benefit [146].

### 4.2 Thiazolidinediones

**FIGURE 3:**
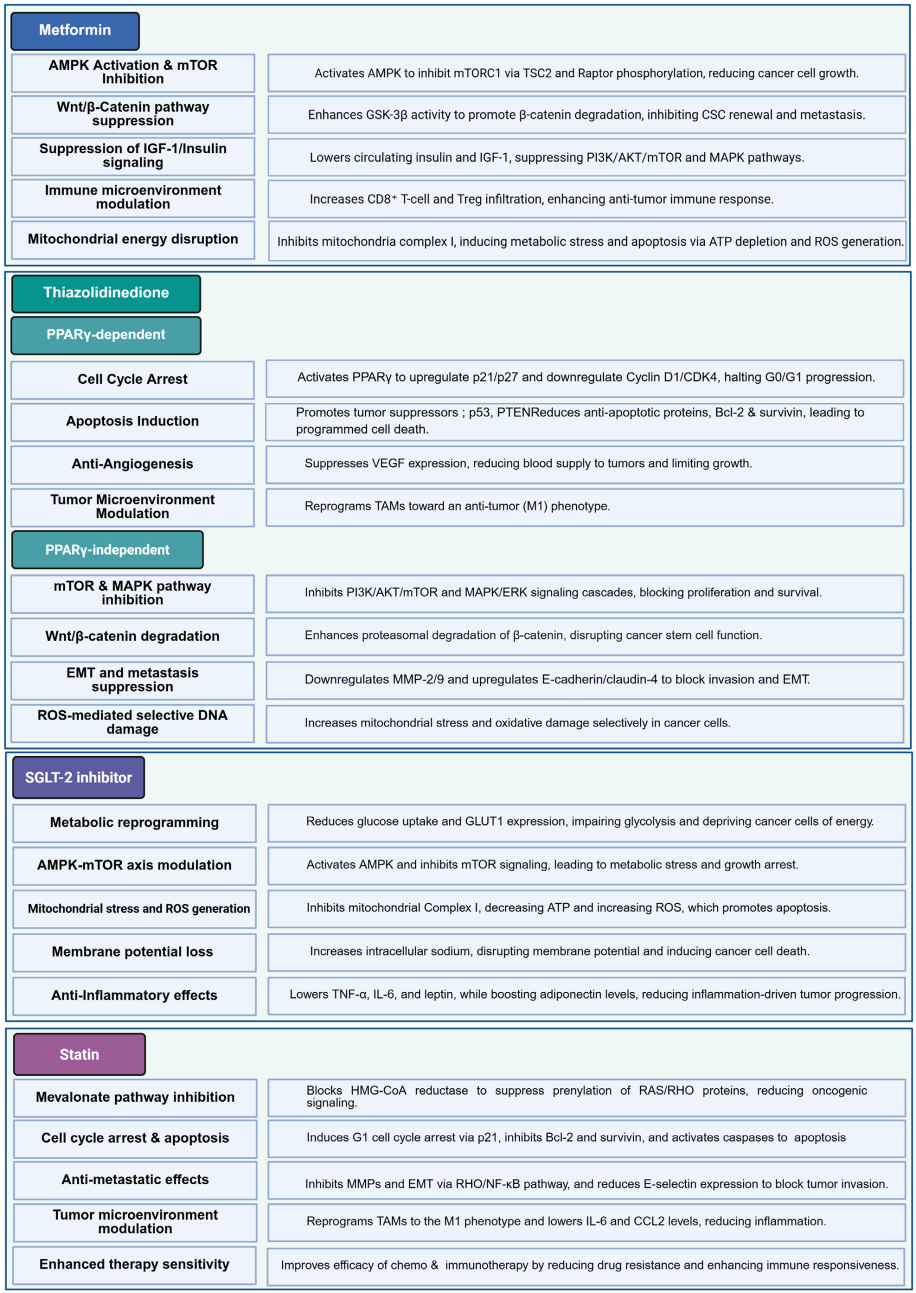
Summary of the mechanisms of action of anti‐diabetic and anti‐dyslipidemia drugs in HNC. This figure presents major action of metformin, thiazolidinedione, SGLT‐2 inhibitor, and statin in head and neck cancer. This figure was created with BioRender.com. AKT, protein kinase B; AMPK, AMP‐activated protein kinase; ATP, adenosine triphosphate; CCL2, CC chemokine ligand 2; CDK, cyclin‐dependent kinase; CSC, cancer stem cell; EMT, epithelial‐mesenchymal transition; GLUT, glucose transporter; GSK‐3β, glycogen synthase kinase‐3β; HMG‐CoA, 3‐hydroxy‐3‐methylglutaryl coenzyme A; IGF‐1, insulin like growth factor‐1; IL‐6, interleukin‐6; MAPK, mitogen‐activated protein kinase; MMP, matrix metalloproteinase; mTORC1, m‐TOR, mammalian target of rapamycin complex 1; PI3K, phosphoinositide 3‐kinase; PTEN, phosphatase and tensin homolog deleted on chromosome 10; ROS, reactive oxygen species; TAMs, tumor‐associated macrophages; TNF‐α, tumor necrosis factor‐alpha; TSC2, tuberous sclerosis complex 2; VEGF, vascular endothelial growth factor.

Thiazolidinediones (TZDs), including pioglitazone and rosiglitazone, are primarily known as peroxisome proliferator‐activated receptor gamma (PPARγ) agonists used for managing type 2 diabetes [163]. However, increasing evidence suggests that they exert significant anti‐cancer effects through both PPARγ‐dependent and PPARγ‐independent mechanisms [21, 164]. PPARγ‐dependent mechanisms of TZDs contribute to cancer suppression by inducing cell cycle arrest, apoptosis, and inhibiting angiogenesis. They promote G0/G1 phase cell cycle arrest by upregulating p27Kip1 and p21 while downregulating cyclin D1 and cyclin‐dependent kinase 2/4 (CDK2/CDK4), thereby preventing cancer cell proliferation. TZDs also induce apoptosis through the activation of p53 and phosphatase and tensin homolog deleted on chromosome 10 (PTEN), while suppressing anti‐apoptotic proteins such as Bcl‐2 and survivin, making cancer cells more susceptible to programmed cell death. Furthermore, TZDs inhibit tumor angiogenesis by reducing VEGF expression, which restricts the tumor's blood supply and limits its growth and metastatic potential. Additionally, they regulate cancer stem cell behavior by modulating the Wnt/β‐catenin signaling pathway and alter the tumor microenvironment by inhibiting NF‐κB activity and shifting tumor‐associated macrophages toward an anti‐tumor phenotype [21, 165, 166, 167, 168] (Figure 3).

Beyond their PPARγ‐dependent effects, TZDs exhibit anti‐cancer properties through various PPARγ‐independent pathways. One of these mechanisms is the inhibition of key oncogenic pathways, including the PI3K/AKT/mTOR and MAPK/ERK signaling cascades. By suppressing these pathways, TZDs significantly reduce tumor cell proliferation, survival, and invasion. They also disrupt the function of anti‐apoptotic proteins such as Bcl‐2 and Bcl‐xL, particularly in response to treatment with troglitazone and ciglitazone, leading to increased mitochondrial apoptosis in cancer cells. Moreover, TZDs facilitate the ubiquitin‐dependent proteasomal degradation of several oncogenic proteins, including cyclin D1 and β‐catenin, further contributing to cancer cell growth inhibition and apoptosis [21, 167, 169] (Figure 3).

Another key mechanism by which TZDs inhibit cancer progression is through their effect on MMPs and EMT. By downregulating MMP‐2 and MMP‐9, TZDs prevent extracellular matrix degradation, which is essential for tumor invasion and metastasis. At the same time, they upregulate E‐cadherin and claudin‐4, which help to maintain epithelial cell adhesion and inhibit EMT, a critical step in cancer metastasis. Additionally, some studies suggest that TZDs, particularly troglitazone, may induce selective DNA damage in cancer cells by increasing mitochondrial stress, leading to apoptosis while sparing normal cells. This suggests a role in ROS modulation, which may contribute to their tumor‐suppressive effects (Figure 3). Several studies have explored the potential association between TZDs and HNC (Table 1). Govindarajan et al. conducted a retrospective cohort study and found that patients exposed to TZDs had a significantly lower risk of developing HNC compared to those not exposed. This risk reduction remained significant when combined with other oral antidiabetic agents but was not observed when TZDs were used alongside insulin [155]. Frohlich et al. demonstrated that TZDs had a chemopreventive effect, reducing the incidence of HNC in diabetic patients [156]. Burotto et al. conducted a meta‐analysis and a retrospective cohort study, showing that TZD use was associated with a substantial reduction in the risk of HNSCC. A large population‐based cohort study also found a moderate risk reduction among TZD users, but the findings were limited to diabetic populations, making their applicability to non‐diabetic individuals uncertain [158]. Conversely, Yang et al. reported that prior use of pioglitazone was associated with a slightly increased risk of HNC, with regular use showing a similar trend, while irregular use was not significant. Among cancer subtypes, oral cavity cancer had a significantly higher risk, whereas no significant associations were found for salivary gland or nasopharyngeal cancer [157].

### 4.3 SGLT‐2 Inhibitors

SGLT‐2 inhibitors, a class of antidiabetic drugs, have recently garnered attention for their potential anticancer effects [170, 171]. While originally designed to control blood glucose levels in type 2 diabetes, these inhibitors may also modulate tumor metabolism, proliferation, and survival through various molecular mechanisms. Cancer cells exhibit high glucose uptake to fuel their rapid proliferation, often utilizing the Warburg effect, where glycolysis is preferred even under normoxic conditions. SGLT‐2 inhibitors interfere with this process by reducing intracellular glucose availability, thereby inhibiting glycolysis and depriving cancer cells of essential energy sources. They also block mitochondrial respiration, as seen in the case of canagliflozin, which inhibits complex‐I of the electron transport chain, leading to impaired ATP production and suppressed cell growth. Furthermore, SGLT‐2 inhibitors can downregulate GLUT1, an essential glucose transporter, which further limits glucose uptake and inhibits proliferation [172]. SGLT‐2 inhibitors also induce apoptosis and mitochondrial dysfunction, leading to cancer cell death. They disrupt mitochondrial function by inducing mitochondrial membrane instability, which was observed in breast cancer models treated with ipragliflozin, resulting in increased mitochondrial membrane depolarization and loss of cell viability. In addition, inhibition of SGLT‐2 leads to increased intracellular sodium levels, causing cell membrane hyperpolarization, which disrupts ionic balance and triggers apoptosis [173]. These inhibitors also activate the AMPK/mTOR pathway, where the reduction in glucose uptake activates AMPK, a cellular energy sensor that downregulates mTOR, leading to growth arrest and metabolic stress [172] (Figure 3). Beyond direct effects on metabolism and apoptosis, SGLT‐2 inhibitors also exhibit anti‐inflammatory properties, which may help suppress tumorigenesis. These drugs have been shown to reduce the levels of inflammatory cytokines such as TNF‐α, IL‐6, and leptin, while increasing adiponectin, leading to a reduction in systemic inflammation. Additionally, they improve adipose tissue function, enhancing insulin sensitivity and metabolic health, which reduces obesity‐driven inflammatory signaling—a key factor in cancers such as breast, colorectal, and pancreatic cancer [172].

The anticancer effects of SGLT‐2 inhibitors vary depending on cancer type. In breast cancer, these inhibitors have been shown to attenuate proliferation via membrane hyperpolarization and mitochondrial dysfunction [173]. In lung cancer, particularly non‐small cell lung cancer (NSCLC), SGLT‐2 inhibitors have been associated with reduced tumor growth and increased survival [174]. Prostate and pancreatic cancers express SGLT‐2, and inhibition leads to a decrease in glucose uptake and cell survival. In liver cancer, canagliflozin has demonstrated the ability to reduce tumor formation by modulating oxidative stress and metabolic reprogramming [172, 175].

While the association between SGLT‐2 inhibitors and various cancers has been reported in multiple studies, research on their relationship with HNC remains limited (Table 1). One study reported that while SGLT‐2 inhibitors were linked to a significantly lower risk of nasopharyngeal carcinoma (NPC) than other anti‐diabetic drug dipeptidyl peptidase 4 (DPP‐4) inhibitors, their association with overall HNC was not statistically significant [159]. Therefore, the role of SGLT‐2 inhibitors in HNC remains unproven, and further research is needed.

## 5 Regulation of Impaired Lipid Profiles in HNC

### 5.1 Statin

Statins, widely recognized for their cholesterol‐lowering properties [176], have demonstrated significant potential as anticancer agents through various molecular mechanisms. These effects are primarily mediated by their ability to inhibit the mevalonate pathway, which is crucial for cancer cell survival and proliferation. The primary anticancer mechanism of statins is the inhibition of 3‐hydroxy‐3‐methylglutaryl coenzyme A (HMG‐CoA) reductase, the rate‐limiting enzyme in the mevalonate pathway. This pathway plays a key role in synthesizing cholesterol and isoprenoids, which are essential for cell signaling and tumor growth. By blocking this pathway, statins reduce the production of farnesyl pyrophosphate (FPP) and geranylgeranyl pyrophosphate (GGPP), which are necessary for the post‐translational modification prenylation of proteins such as RAS, RHO(RAS Homologous), RAC(RAS‐related C3 botulinum toxin substrate), and RAP(RAS‐related protein). These proteins are critical for oncogenic signaling, and their inhibition disrupts cancer cell proliferation and survival [177, 178, 179].

Statins have been shown to promote cancer cell apoptosis through multiple pathways. They activate caspase‐3, −7, and −9, leading to programmed cell death. Additionally, they inhibit anti‐apoptotic proteins like Bcl‐2 and survivin, which disrupt mitochondrial membrane integrity and trigger apoptosis. Moreover, statins induce cell cycle arrest at the G1 phase by suppressing cyclin D1 and upregulating p21, effectively halting tumor progression [179]. Statins impair cancer cell migration and metastasis by inhibiting the RhoA/Rho kinase/NF‐κB pathway, a key regulator of cellular motility. They also suppress EMT, a critical process in metastatic progression, and prevent tumor cell adhesion to endothelial cells by reducing E‐selectin expression, thereby limiting the metastatic potential of cancer cells. Statins reshape the tumor microenvironment (TME) by reprogramming tumor‐associated macrophages (TAMs) from a tumor‐promoting M2 phenotype to an antitumor M1 phenotype, which enhances immune responses against cancer cells. Additionally, statins reduce the secretion of pro‐inflammatory cytokines such as IL‐6 and CC chemokine ligand 2 (CCL2), thereby mitigating inflammation‐driven tumor progression. They also disrupt cancer cell metabolism by reducing lactic acid production, limiting the survival of hypoxic tumor cells. Statins exhibit anti‐angiogenic properties by inhibiting VEGF signaling, which suppresses tumor vascularization. They also block basic fibroblast growth factor (bFGF) and hepatocyte growth factor (HGF) signaling, further preventing blood vessel formation in tumors and reducing their ability to obtain oxygen and nutrients [179, 180]. Statins have been shown to enhance the efficacy of chemotherapeutic agents by reducing multidrug resistance (MDR), particularly through the inhibition of drug efflux pumps such as P‐glycoprotein. They also potentiate the effects of chemotherapeutic agents like cisplatin, doxorubicin, and fluorouracil by promoting cancer cell apoptosis. Additionally, emerging research suggests that statins may improve responses to immune checkpoint inhibitors (PD‐1 inhibitors), particularly in cancers such as lung cancer and melanoma [179]. Apart from apoptosis, statins trigger other forms of programmed cell death. They induce autophagic cell death, which reduces cancer cell survival, and promote ferroptosis, an iron‐dependent cell death mechanism, by inhibiting the Glutathione/Glutathione Peroxidase 4 (GSH/GPX4) pathway, leading to increased lipid peroxidation and oxidative stress in cancer cells [178, 179].

Meta‐analyses suggest that statin use is associated with a lower risk and improved prognosis in gynecologic cancers such as endometrial and ovarian cancer. In colorectal cancer, studies indicate a modest but statistically significant protective effect, particularly in reducing metastasis. In breast and prostate cancers, high‐dose statins may enhance chemotherapy efficacy and reduce tumor proliferation. While preclinical and clinical data are promising, further well‐controlled, large‐scale clinical trials are necessary to confirm the therapeutic potential of statins in oncology [179]. Several studies have investigated the relationship between statin use and HNC with mixed but generally positive findings (Table 1). Research suggests that post‐diagnosis statin use may improve survival, with one study reporting a 22% reduction in all‐cause mortality (HR = 0.78, 95% CI: 0.67–0.90, *p* = 0.001) [160]. Additionally, a systematic review found that 75% of HNC‐related studies indicated a protective effect of statins, with one study showing a 28% lower risk of recurrence (OR = 0.72, 95% CI: 0.58–0.89, *p* = 0.003) [161]. Long‐term statin use (more than 2 years) was associated with a 17% reduced risk of developing HNC (OR = 0.83, 95% CI: 0.75–0.91, *p* < 0.001), while short‐term use (≤ 1 year) showed no significant effect [162].

Statins exhibit multifaceted anticancer mechanisms, including inhibition of the mevalonate pathway, induction of apoptosis and cell cycle arrest, suppression of metastasis and invasion, modulation of the tumor microenvironment, anti‐angiogenic effects, sensitization to chemotherapy, and induction of autophagy and ferroptosis (Figure 3). Given their broad spectrum of anticancer activities and established safety profile, statins hold significant promise as repurposed drugs for cancer treatment. However, further clinical research is required to optimize their use as stand‐alone or combination therapies in oncology [178, 179, 181].

## 6 Discussion

The relationship between MetS and HNC is an emerging area of interest in oncology, yet current evidence remains inconclusive. While several studies suggest that MetS may increase HNC risk, others indicate that individual components of MetS—rather than the syndrome as a whole—may be the primary drivers of carcinogenesis. For example, obesity has been associated with both increased and decreased HNC risk, suggesting that its influence on tumorigenesis is complex and potentially dependent on factors such as tumor subtype, anatomical location, and systemic metabolic alterations. Similarly, diabetes and dyslipidemia have been implicated in promoting tumor progression via chronic inflammation, insulin resistance, and lipid dysregulation, yet conflicting epidemiological data underscore the need for further investigation into their precise oncogenic roles (Figure 1).

Among pharmacologic interventions, metformin has been the most extensively studied for its potential anti‐cancer properties. As an AMPK activator and mTOR inhibitor, metformin exerts tumor‐suppressive effects by reducing cancer cell proliferation, enhancing apoptosis, and modulating metabolic signaling (Figure 2). Preclinical and retrospective clinical studies suggest that metformin may lower HNC incidence, enhance radiosensitivity, and improve survival, particularly in HPV‐negative, tobacco‐associated HNC subtypes. However, conflicting clinical data indicate that metformin's efficacy may vary based on tumor molecular characteristics, HPV status, and patient‐specific metabolic conditions. Given these discrepancies, large‐scale randomized controlled trials are essential to determine its precise therapeutic role, optimal dosing strategies, and potential synergies with existing treatment modalities.

TZDs, including pioglitazone, have demonstrated anti‐proliferative and pro‐apoptotic effects via PPARγ activation, cell cycle arrest, and inhibition of angiogenesis (Figure 3). Some studies suggest a chemopreventive role in certain malignancies, but their application in HNC remains poorly defined. Notably, concerns have been raised regarding the potential oncogenic risks associated with long‐term TZD use, particularly in relation to bladder cancer. The lack of mechanistic studies and clinical trials evaluating their role in HNC precludes definitive conclusions, underscoring the need for further research into their safety, efficacy, and mechanisms of action in this specific cancer type.

SGLT‐2 inhibitors have emerged as potential metabolic modulators in oncology, with preclinical data suggesting that they reduce intracellular glucose availability, induce mitochondrial dysfunction, and suppress inflammation, thereby impairing cancer cell survival (Figure 3). However, research on SGLT‐2 inhibitors in HNC is extremely limited, with only one study reporting a potential protective effect against nasopharyngeal carcinoma. Their role in other HNC subtypes remains unknown, and no robust clinical trials have been conducted to assess their efficacy as adjunctive therapy. Given their ability to alter metabolic homeostasis and impact tumor energy metabolism, further in vitro, in vivo, and clinical investigations are warranted to determine their potential oncologic applications.

Statins, widely used in the management of dyslipidemia and cardiovascular disease, have been studied for their anti‐cancer properties, primarily through inhibition of the mevalonate pathway, suppression of oncogenic protein prenylation, and induction of apoptosis (Figure 3). Epidemiologic studies indicate that long‐term statin use may be associated with a reduced risk of HNC and improved survival outcomes, particularly when used post‐diagnosis. However, other analyses report no significant benefit or, in some cases, an increased cancer risk, suggesting that statin effects may be context‐dependent and influenced by patient metabolic status, tumor subtype, and concurrent therapies. Further research is necessary to determine which patient populations may derive the greatest benefit from statin therapy and whether these agents can be effectively integrated with chemotherapy, radiotherapy, or immunotherapy.

Among metabolic‐targeting agents, metformin has shown the greatest potential in HNC prevention and treatment, but its efficacy appears to be influenced by tumor subtype, patient metabolic status, and treatment modality. While TZDs and SGLT‐2 inhibitors exhibit promising anti‐cancer mechanisms, their application in HNC remains underexplored, necessitating further preclinical and clinical research. Statins may provide therapeutic benefits, but findings remain inconsistent, emphasizing the need for well‐designed trials to clarify their role in HNC management.

Here, we reviewed the potential use of various metabolic modulating agents as first‐line or adjuvant therapy for HNC. Further in vitro and in vivo studies to elucidate the intracellular mechanisms are essential before these agents can be actively used in the clinic as therapeutic and preventive therapies for HNC, and these studies should provide well‐established guidelines for the prevention and treatment of HNC in people with MetS.

## Author Contributions

**Sujung Yeom:** conceptualization, writing – original draft, visualization. **Dong Hoon Lee:** conceptualization, writing – original draft, visualization, funding acquisition, supervision, writing – review, and editing. **Juhyun Song:** conceptualization, writing – original draft, visualization, funding acquisition, supervision, writing – review, and editing. All authors read and approved the final manuscript.

## Conflicts of Interest

The authors declare no conflicts of interest.

## Data Availability

The authors have nothing to report.
